# Licorice‐Induced Pseudohyperaldosteronism: A Case Report

**DOI:** 10.1002/ccr3.70005

**Published:** 2025-01-12

**Authors:** David D. Je, Yong Mong Tan, Gradon Fuller, Parul Nigam

**Affiliations:** ^1^ Townsville Hospital and Health Service Townsville Queensland Australia; ^2^ College of Medicine and Dentistry James Cook University Townsville Queensland Australia

## Abstract

We present a 71‐year‐old man admitted to the intensive care unit with severe hypokalemia after ingesting licorice‐containing confectionary over 6 weeks. Excess consumption of licorice with its active metabolite glycyrrhizic acid may precipitate pseudohyperaldosteronism with life‐threatening hypokalemia. Public awareness and measures such as warning labels are needed to warn against excessive consumption.

## 1 Background

Licorice ( *Glycyrrhiza glabra* ) root is a plant that is widely known as a traditional therapeutic (anti‐ulcer, antispasmodic, anti‐inflammatory, anti‐oxidative, antimicrobial, anticarcinogenic, etc.) as well as a popular sweetener. It contains an active metabolite glycyrrhizic acid which inhibits a renal enzyme 11 beta‐hydroxysteroid dehydrogenase type 2 (11‐beta‐HSD2). In excess, this can result in toxicity, manifesting as pseudohyperaldosteronism, a rare complication of licorice ingestion with mainly case reports in the literature to date. We describe a case of licorice‐induced pseudohyperaldosteronism presenting as severe hypokalemia after a significant subacute ingestion of licorice‐containing confectionary.

## 2 Case Presentation

A 71‐year‐old Caucasian man was referred by his cardiologist to the emergency department of a local hospital for hypokalemia of 2.1 mmol/L during preadmission investigations prior to an elective coronary angiogram for stable angina with positive computed tomography coronary angiogram. Over the past month, he also had intermittent palpitations, but was otherwise in his usual state of health with no dyspnoea, change in exercise tolerance, or muscle weakness. He denied any nausea, vomiting, diarrhea, or change in appetite or weight.

His medical history was significant for hypertension, dyslipidaemia, ischaemic heart disease with previous coronary artery stenting in 2011, gout, obstructive sleep apnoea on continuous positive airway pressure therapy. His regular medications were daily telmisartan 80 mg daily, hydrochlorothiazide 12.5 mg, amlodipine 5 mg, atenolol 50 mg, aspirin 100 mg, allopurinol 300 mg, rosuvastatin 40 mg, and levothyroxine 50 mcg. There was no family history of adrenal disease or diabetes mellitus. He drank approximately 6 standard drinks of alcohol daily and did not smoke cigarettes. On further history, it was noted that he had ingested approximately 800 g of licorice confectionary (raw licorice content of 30.4 g) over the preceding 6 weeks which his daughter gifted to him for his birthday.

On physical examination, his Glasgow Coma Scale was 15, blood pressure (BP) was 164/73 mmHg (the last BP measurement with his general practitioner 5 months prior was 130/78 mmHg), heart rate was 45 beats per minute and regular, saturations were normal in room air, and he was afebrile. Body mass index was 38 kg/m^2^ (111.6 kg, 172 cm). Neurological examination was normal. Heart sounds were dual with no murmur and lungs were clear on auscultation. There was mild pitting ankle oedema which he reported was longstanding. Abdominal examination was normal and there were no overt Cushingoid features.

## 3 Investigation

Initial investigations on admission to hospital showed serum potassium of 2.3 mmol/L which dropped to a nadir of 1.9 mmol/L on Day 2; his last serum potassium nearly 8 weeks prior to admission was normal at 4.2 mmol/L. Serum sodium was 148 mmol/L, chloride 97, bicarbonate 36 mmol/L, pH 7.53, venous pCO_2_ of 57 mmHg, venous pO_2_ of 37 mmHg, corrected calcium of 2.39 mmol/L, phosphate of 0.74 mmol/L, magnesium 0.86 mmol/L, creatinine of 104 umol/L, and eGFR of 62 mL/min/1.73m^2^.

Other investigations during this admission were creatinine kinase (CK) 1593 U/L (reference: 40–200) and high sensitivity troponin 56 ng/L and 64 ng/L on repeat testing (reference: < 26 ng/L). Electrocardiogram (ECG) showed sinus bradycardia with flattened T waves in lateral leads, T wave inversion in lead III, but no QT segment prolongation.

Random urine osmolality was 489 mmol/kg (reference: 50–1450 mmol/kg), creatinine 4.0 mmol/L, sodium 90 mmol/L, potassium 50 mmol/L, chloride 109 mmol/L, with paired serum sodium 144 mmol/L, potassium 2.2 mmol/L, creatinine 89 umol/L, and chloride 94 mmol/L.

Serum cortisol at 11 a.m. was 333 (reference: 100–535 nmol/L). 24‐h urinary free cortisol (LC–MS/MS) was 626 nmol/d (reference: < 130 nmol/d). Seated serum aldosterone was 50 pmol/L (reference: 100–950 pmol/L), renin < 2.0 mU/L (reference: 3.3–41 mU/L), aldosterone: renin ratio < 25 (reference: < 70), and DHEAS 1.2 umol/L (reference: 0.75–7.70 umol/L).

## 4 Treatment

Hydrochlorothiazide was ceased on admission. The patient was admitted to the intensive care unit (ICU) for intensive potassium replacement through a peripherally inserted central catheter (PICC) line with cardiac telemetry monitoring.

Spironolactone was commenced at 50 mg twice daily on Day 4 of admission for persistent hypokalemia of 3.7 mmol/L despite aggressive IV potassium of approximately 800 mmol to date. On the same day, prednisolone 40 mg daily was also commenced for symptomatic flare of gout in the left knee with satisfactory relief of symptoms.

## 5 Outcome and Follow‐Up

By Day 6 of admission, potassium was stable at 4.0 mmol/L on oral potassium 1800 mg four times daily and he was discharged from the ICU to the ward. He was discharged from the hospital on Day 9 of admission with potassium of 4.0 mmol/L, on telmisartan 40 mg daily, spironolactone 50 mg twice daily, prednisolone 40 mg daily, and no potassium supplementation. Table 1 shows the investigations and management from the day of admission to hospital to discharge. He was advised to cease licorice‐containing confectionary.

**TABLE 1:** Investigations and management from day of admission to discharge.

Day of admission	Investigations	Management
Day 1	Sodium 148 mmol/L Potassium 2.3 mmol/L (ref: 3.5–5.5 mmol/L) Chloride 97 Bicarbonate 36 mmol/L pH 7.53 Venous pCO_2_ 57 mmHg Venous pO_2_ 37 mmHg Corrected calcium 2.39 mmol/L Phosphate 0.74 mmol/L Magnesium 0.86 mmol/L Creatinine 104 umol/L eGFR 62 mL/min/1.73m^2^ Creatinine kinase 1593 U/L (ref: 40–200) High sensitivity troponin 56 ng/L which was 64 ng/L on repeat testing (ref: < 26 ng/L) ECG: sinus bradycardia with flattened T waves in lateral leads, T wave inversion in lead III	Hydrochlorothiazide ceased on admission ICU admission for intensive potassium replacement
Day 2	Urine biochemistry: Osmolality 489 mmol/kg (ref: 50–1450 mmol/kg) Sodium 90 mmol/L Potassium 50 mmol/L Chloride 109 mmol/L Creatinine 4.0 mmol/L Paired serum biochemistry: Sodium 144 mmol/L Potassium 2.2 mmol/L Chloride 94 mmol/L Creatinine 89 umol/L Random cortisol (11 am) 333 (ref: 100–535 mmol/L)	
Day 3	Seated aldosterone 50 pmol/L (ref: 100–950 pmol/L) Renin < 2.0 mU/L (ref: 3.3–41 mU/L) Aldosterone:renin ratio < 25 (ref: < 70) DHEAS 1.2 umol/L (ref: 0.75–7.70 umol/L) HbA1c 5.5% (< 6.5%)	
Day 4	Potassium 3.7 mmol/L	Spironolactone commenced at 50 mg twice daily Prednisolone 40 mg daily commenced for left knee gout flare
Day 6	Potassium 4.0 mmol/L	Discharged from ICU to ward
Day 9	Potassium stable at 4.0 mmol/L	Discharged with potassium of 4.0 mmol/L, on telmisartan 40 mg daily, spironolactone 50 mg twice daily, prednisolone 40 mg daily, no potassium supplementation.

He was followed up in the endocrine clinic and spironolactone and prednisolone were weaned off with monitoring of BP and potassium, aldosterone, and renin levels.

## 6 Discussion

Licorice has a variety of health benefits which including: (a) use in Chinese herbal medicine to treat gastric ulcers, (b) improvement in postural hypotension due to diabetic autonomic neuropathy, (c) reduction in serum potassium concentration, (d) some efficacy in treating chronic hepatitis B, (e) reduction in fat mass, and (f) antitumor effects with dose dependent effect on tumor proliferation and apoptosis [1]. Of relevance to our patient who has ischemic heart disease, in vitro and in vivo studies demonstrated anti‐inflammatory, anti‐cytokine, antioxidant, antiatherogenic, and anti‐placelet effects of licorice [2].

However, licorice consumption has known risks, including pseudohyperaldosteronism. Cortisol is a potent stimulator of the mineralocorticoid receptor, which in normal glucocorticoid metabolism, is converted to cortisone by 11 beta‐hydroxysteroid dehydrogenase type 2 (11‐beta‐HSD2) in the kidneys. Glycyrrhizic acid and its aglycone glycyrrhetinic acid are constituents of licorice that inhibit 11‐beta‐HSD2 that can cause an increased concentration of cortisol to activate the mineralocorticoid receptor [3]. This excess mineralocorticoid receptor activation then leads to pseudohyperaldosteronism, which clinically mimics hyperaldosteronism but with suppression of both aldosterone and renin levels. Other than dietary, pseudohyperaldosteronism can be classified into genetic and endocrinal causes [1]. In the case of primary hyperaldosteronism due to adrenal hypersecretion of aldosterone, the plasma renin level is low, whereas secondary hyperaldosteronism is caused by excessive activation of the renin–angiotensin–aldosterone system (RAAS) due to renal artery stenosis or oedematous disorders [4].

Our patient's hypokalemia could have been exacerbated by his thiazide diuretic and clinically other than mild palpitations he had no other symptoms attributable to pseudohyperaldosteronism. His clinical and biochemical features were hypertension, nonspecific ST and T wave changes on ECG, severe hypokalemia, hypernatremia, metabolic alkalosis, suppressed aldosterone and renin levels, and CK rise without clinical myopathy. There is no definitive diagnostic criterion for licorice‐induced pseudohyperaldosteronism, but rather, it is on the basis of the clinical presentation with a recent history of licorice intake. Pseudohyperaldosteronism due to licorice toxicity can mimic the features of primary aldosteronism without elevation of serum aldosterone concentration: electrolyte abnormalities may include hypokalemia, hypernatraemia, metabolic alkalosis, and acute kidney injury; neuromuscular manifestations can include paralysis, epileptiform manifestations, altered level of consciousness, rhabdomyolysis, and elevated CK level; cardiovascular effects can include ECG changes such as QT interval prolongation, U waves, slight ST segment elevation in V1–V3, supraventricular tachycardia, as well as hypertension, heart failure, pulmonary oedema, and cardiac death (two cases) [5]. In addition, studies showed expression of mineralocorticoid receptor and 11‐beta‐HSD1/2 in the heart and arteries, and inhibition of 11‐beta‐HSD2 increased the contractile response to phenylephrine and altered the relaxation of arteries [6]. In light of the potential deleterious side effects of licorice, deglycyrrhizinated licorice (DGL) has been developed, and example of which is the “Chewable DGL by bioclinic natural” available in Australia. However, components of licorice apart from glycyrrhizin can also confer health risks, such as the potentiation of certain drugs through CYP3A4 inhibition (e.g., warfarin and ciclosporin) [1].

Our patient had an elevated 24 h urinary free cortisol level without clinical features of Cushing's syndrome. Ingestion of glycyrrhetinic acid has been shown to cause elevation of urinary excretion of free cortisol, virtually unchanged plasma cortisol, and decreased plasma cortisone and urinary free cortisone, because of inhibition of conversion of cortisol to cortisone [7]. A high urinary cortisol–cortisone ratio is suggestive of 11‐beta‐HSD2 inhibition [8], though this was not performed in our patient. Licorice consumption has also been shown to elevate salivary cortisol levels [9]. Our patient's urinary free cortisol was followed and was normal to ensure recovery after a period of licorice cessation.

Our patient consumed approximately 30.4 g of raw licorice over 6 weeks, that is, 720 mg/day. The content of glycyrrhizin of licorice was unknown in our case, but in general varies from 2%–25% [1]. This equates to 14.4–180 mg of glycyrrhizin per day which in our patient exceeded the maximum recommended daily intake. The Scientific Committee on Food in 2003 concluded that an upper limit for regular consumption of 100 mg/d provides sufficient protection for the majority of the population [10]. The World Health Organization also determined that glycyrrhizin intake of 100 mg/day (approximately 2 mg/kg) was unlikely to cause adverse effects in the majority of adults [11].

Clinical risk factors for pseudohyperaldosteronism in our patient was his older age and concomitant intake of a thiazide diuretic for hypertension. Daily dosage and long‐term consumption of licorice, female sex, older age, and comorbidities such as preexisting hypertension, kidney disease, electrolyte disturbance (i.e., hypokalemia, hypernatraemia, metabolic alkalosis), constipation, hypoalbuminaemia, and hyperbilirubinaemia can predispose to developing pseudohyperaldosteronism [5, 6]. Those with preexisting cardiovascular disease are at higher risk of major adverse cardiovascular events (MACEs); diarrheal and vomiting diseases could predispose to electrolyte disturbance; polycystic ovarian syndrome patients are more prone to hypertension [5]. Systemic glucocorticoids that can aggravate the inhibition of 11‐beta‐HSD2, and antihypertensives (potassium‐losing and potassium‐sparing) can affect the phenotype of licorice‐induced pseudohyperaldosteronism [5].

Our patient received aggressive potassium replacement after hydrochlorothiazide and licorice were ceased. He received an average of 267 mmol IV potassium per day for the first 3 days to reach achieve a serum level of 3.7 mmol/L from a nadir of 1.9 mmol/L. In almost all reported cases, the clinical manifestations improved with licorice cessation and electrolyte replacement, though hospitalization was common, occurring in 84 out of 104 cases [5]. The lingering effects of his hydrochlorothiazide with its elimination half‐life of 6–12 h [12] contributed to the high potassium requirement. The clinical recovery timeframe of licorice‐induced hyperaldosteronism varies and may take up to 6 months for the renin–angiotensin–aldosterone axis to normalize, owing to the long half‐life of glycyrrhetic acid [13].

Spironolactone was commenced on admission Day 4. Aldosterone receptor antagonism with spironolactone or eplerenone has been shown to normalize vascular function and BP in licorice‐induced hypertension in animal models and may play a role in treatment [14]. Dexamethasone was considered, though never commenced because of the concurrent use of prednisolone for acute gout flare‐up. Dexamethasone can be considered in severe hypokalemia as it suppresses the endogenous production of cortisol [15] without appreciably activating the mineralocorticoid receptor [16]. On the other hand, administration of glucocorticoids with mineralocorticoid activity (e.g., prednisolone) can induce pseudohyperaldosteronism by binding glucocorticoid and/or mineralocorticoid receptors [17].

Though recognized as generally safe, toxicity becomes more likely above recommended levels and the public health concern of licorice in food items in food remains topical. The quantity limits of licorice content as per the U.S. Food and Drug Administration varies from 0.05% of glycyrrhizin in baked goods to 16.0% of glycyrrhizin in hard candy [18]. The glycyrrhizin content of food items are often not specified beyond licorice content, which can have varying percentages of glycyrrhizin and there is generally no warning regarding toxicity. Thus, it is difficult for the consumer to be aware of, and mitigate, the risks. Overall, given that the complication of licorice‐induced pseudohyperaldosteronism, including severe hypokalemia, can be a life‐threatening emergency, it should be a public health safety requirement for manufacturers of high content licorice food items to caution excessive consumption on its label and specify a daily consumption limit for the product.

In conclusion, a history of licorice intake should be considered in anyone presenting with hypokalemia. Large amounts of potassium replacement may be required for the hypokalemia caused by licorice‐induced pseudohyperaldosteronism. The clinical manifestation of licorice‐induced pseudohyperaldosteronism may be exacerbated by various factors. Increased public awareness of the serious health risk of excess licorice consumption is required.

## Author Contributions

**David D. Je:** data curation, formal analysis, funding acquisition, investigation, methodology, project administration, writing – original draft, writing – review and editing. **Yong Mong Tan:** data curation, formal analysis, funding acquisition, investigation, methodology, project administration, supervision, writing – original draft, writing – review and editing. **Gradon Fuller:** data curation, formal analysis, investigation, methodology, writing – original draft, writing – review and editing. **Parul Nigam:** conceptualization, data curation, formal analysis, funding acquisition, investigation, methodology, project administration, supervision, writing – original draft, writing – review and editing.

## Consent

Written informed consent for publication of their clinical details and/or clinical images was obtained from the patient.

## Conflicts of Interest

The authors declare no conflicts of interest.

## Data Availability

All data underlying the results are available as part of the article and no additional source data are required.
