# Correction to Licorice‐Induced Pseudohyperaldosteronism: A Case Report

**DOI:** 10.1002/ccr3.70594

**Published:** 2025-07-16

Je, D. D., Tan, Y. M., Fuller, G., & Nigam, P. (2025). Licorice‐Induced Pseudohyperaldosteronism: A Case Report. *Clinical Case Reports*, *13*(1), e70005. https://doi.org/10.1002/ccr3.70005

The funding statement for this article was missing. The below funding statement has been added to the article:

The study was supported by THHS Study, Education and Research Trust Account funds (SERTA).

We apologize for this error.

